# Safety and effectiveness of ferric carboxymaltose intravenous therapy in pediatric patients with chronic kidney disease

**DOI:** 10.3389/fped.2022.967233

**Published:** 2022-10-06

**Authors:** Patricia Garcia-Ortega, Ines Jimenez-Lozano, Álejandro Cruz, Aurora Fernandez Polo, Mercedes Lopez, Gema Ariceta

**Affiliations:** ^1^Department of Pharmacy, Vall d'Hebron University Hospital, Barcelona, Spain; ^2^Department of Pediatric Nephrology, Vall d'Hebron University Hospital, Barcelona, Spain; ^3^Department of Pediatrics, Autonomous University of Barcelona, Barcelona, Spain

**Keywords:** pediatrics, kidney disease, intravenous iron, ferric carboxymaltose, iron deficiency, anemia

## Abstract

Iron-deficiency anemia is the most common reason for worsening of the anemia characteristically seen in chronic kidney disease (CKD). Ferric carboxymaltose (FCM) is a macromolecular hydroxide ferric carbohydrate complex that allows high-dose iron to be administered parenterally for gradual, controlled release. The aim of this study was to retrospectively evaluate the safety and effectiveness of FCM treatment in pediatric patients with CKD non-dependent of hemodialysis, seen at a tertiary hospital. Data were collected on demographics, dosage, infusion time, laboratory results, and tolerability of the medicinal product. A total of 79 patients (40.5% girls) were included; the median age [25th percentile (P25) to 75th percentile (P75)] was 9 years (5–13). Laboratory results at 15–45 days post-infusion revealed a median increase of 1.4 g/dL (0.9–1.9) in hemoglobin, 224 μg/L (136–378.5) in ferritin, 37 μg/dL (17.5–71) in serum iron, and 18% (9.3–27.8) in transferrin saturation. All patients tolerated FCM infusions well, and no serious hypersensitivity reactions or anaphylactic reactions were observed. Only one adverse event was identified: drug extravasation at the end of the infusion in a 16-year-old patient. These data provide further evidence for the use of FCM as a safe and effective therapeutic option in pediatric patients with CKD, based on the low incidence of adverse effects, minor intervention required, and anemia improvement based on laboratory results.

## Introduction

Iron-deficiency anemia is the most common reason for worsening of the anemia characteristically seen in chronic kidney disease (CKD). In patients with CKD, decreased erythropoiesis is exacerbated by concomitant conditions of chronic inflammation and the resulting increased production of hepcidin, which inhibits intestinal absorption of

iron and mobilization of iron deposits from the reticuloendothelial system (RES) to circulating transferrin (1). In addition, children experience circulating iron loss due repeated blood draws and during therapeutic procedures, in a larger proportion than adults with similar clinical condition (2).

Consequently, iron deficiency treatment is very common in CKD, and often is associated with epoetin therapy. Selection of the route used to administer iron depends on deficiency severity, venous access availability, prior response to oral therapy, adverse effects (AEs) observed, patient compliance, and cost. The Kidney Disease: Improving Global Outcomes (KDIGO) clinical guidelines for the diagnosis and treatment of anemia in pediatric patients with CKD recommend maintaining transferrin saturation (TS) ≥ 20% and ferritin ≥ 100 ng/mL and starting treatment with oral iron (or intravenous iron during hemodialysis sessions) to maintain these values, even if patients are receiving erythropoiesis-stimulating factors (ESAs) (3, 4).

At early stages of CKD, oral iron therapy has been traditionally recommended and used because it is less invasive and less expensive, despite issues related to gastrointestinal tolerance and absorption are common (5). In addition, more than 2 to 3 weeks are usually needed to detect an increase in hemoglobin (Hb) and up to 6 months to attain adequate iron deposits, resulting in delayed therapeutic response (2, 6–8). Recently, the advent of new intravenous iron preparations with a good safety and tolerability profile (9–11) has made it possible to use higher doses at less frequent intervals. As consequence IV iron use is, increasing among patients with kidney disease who are not on hemodialysis as dose administration can fit to less frequent hospital visits for regular care. Further, IV iron administration allows epoetin optimization as is not influence by patient compliance.

Ferric carboxymaltose (FCM) is a macromolecular hydroxide ferric carbohydrate complex that allows for gradual, controlled release of iron inside RES cells, with uptake by endogenous iron-binding proteins. Because this complex is highly stable, ionic iron is not released directly into the bloodstream, preventing potential cell damage due to oxidative stress (12) and thus allowing high doses to be administered each time: 15 mg of iron/kg by intravenous injection or up to 20 mg of iron/kg by intravenous infusion. Based on drug data sheet, the medication is recommended for the treatment of iron deficiency in pediatric patients older than 14 years or when oral iron preparations are ineffective or cannot be used. According to the summary of product characteristics (SmPC), the most common adverse reactions are nausea, injection/infusion site reactions, low blood phosphate levels, headache, erythema, dizziness, and hypertension (12).

Only a few studies have examined the role of FCM for the treatment of iron-deficiency anemia in pediatric patients, and most have included patients with a gastrointestinal condition.

At our hospital, treatment with intravenous iron in pediatric patients with kidney disease has traditionally been based on the use of iron sucrose and has mainly been limited to patients undergoing hemodialysis, due to the need for repeated doses. The availability of FCM made it possible to extend the use of parenteral iron to patients who do not visit the hospital often, as those not on hemodialysis. Therefore, less effective and tolerated oral iron treatment have been replaced and indeed, clinical practice has shifted considerably toward the intravenous treatment of iron deficiency far more often. The aim of this study was to describe our experience and evaluate the safety and hematologic response of FCM treatment in pediatric patients with kidney disease.

## Materials and methods

The research was designed as a retrospective, observational, single-center study that included all pediatric patients aged 18 years or younger diagnosed with kidney disease who were treated with FCM (Ferinject^®^ Vifor Pharma, St Gallen, Switzerland) between during a 19-month period. Our institution protocol approved that pediatric patient younger than 14 years received FCM as an off-label indication, but after obtaining a prior written consent from parents or guardians. Elegible patients to receive FCM where those who, according to medical criteria, could benefit from of FCM treatment either due to anemia or iron deficiency. Anemia was defined according to the age-based criteria of the KDIGO guidelines: Hb ≤ 11.0 g/dL in children aged 0.5–5 years, Hb ≤ 11.5 g/dL in children aged 5–12 years, Hb ≤ 12 g/dL in children aged 12–15 years, and Hb ≤ 12 g/dL or ≤ 13 g/dL in adolescent girls or boys over 15 years old, respectively; low serum iron deposits was defined as TS ≤ 20% and/or ferritin ≤ 100 ng/mL (3).

Patients were identified using our pediatric hospital e-prescription program for inpatients or outpatients treated at day hospital. In the latter group, FCM infusions coincided with patient scheduled blood draws for lab work and regular follow up visits at the outpatient nephrology clinic of the Nephrology Department. Intravenous administration consisted in diluting FCM in normal saline (50 mL for doses of 100–499 mg; 100 mL for doses of 500–1000 mg) to achieve concentrations equal to or> 2 mg/ml, as indicated in the SmPC for the drug and then infusing the solution over 30 to 60 min.

Electronic medical records were reviewed to collect patients' demographic and clinical data, including age, sex, weight, diagnosis, estimated glomerular filtration rate (eGFR) based on the updated Schwartz formula (12), iron therapy in the previous 6 months, concomitant treatment with ESAs, and type of dialysis. Laboratory results were also collected for Hb, serum iron, ferritin, TS, and blood phosphate before and after FCM infusion. Infusion times and tolerability information were collected from the computer records for each dose.

All patients who received at least 1 dose of FCM during the study period were included in the safety analysis. To evaluate effectiveness, patients were included if data were available for baseline Hb (up to 30 days before FCM administration) and post-administration Hb (at 7–45 days). Changes in Hb were analyzed in patients with increased Hb, and these patients were stratified according to whether or not they received concomitant treatment with ESAs. In patients who received more than 1 infusion of FCM due to low Hb levels after a satisfactory initial response or pre-programmed dose to complete the dosage according to the calculation of Fe requirements, the change in Hb was taken as the first value after the first dose compared with baseline. Patients who showed no increase were studied separately. Changes in the other laboratory results were also analyzed.

This project was approved by the Clinical Research Ethics Committee of our hospital on December 20, 2019 under Protocol Code FCM-NEFRO-PED. Due to the retrospective nature of the study, there were no interventional procedures or changes to current practice in patient management, and all therapeutic decisions were made before the study was undertaken.

The univariate statistical analysis was descriptive. Continuous variables were expressed as the mean and standard deviation, or median and interquartile range (25th percentile-75th percentile). Categorical variables were listed according to absolute number and the respective relative frequencies.

## Results

A total of 79 patients were included; Tables 1, 2 lists the demographic characteristics, treatment administered (number of doses, absolute dosage in mg, dosage in mg/kg, and administration time per dose) and patients CKD stage.

**Table 1:** Biodemographic data.

**Total, n**	**79**
**Sex, n (%)**	
Male	47 (59.5%)
Female	32 (40.5%)
**Age (years)**	
Median (P25–P75)	9 (5–13)
**Weight (kg)**	
Median (P25–P75)	26.4 (18.3–39.5)
**Prior treatment with iron**, ***n*** **(%)**	52 (65.8%)
Oral	21 (40.4%)
IV iron sucrose	30 (57.7%)
IV ferric carboxymaltose	1 (1.9%)
**Ethnicity (%)**	
Caucasian	59 (74.7%)
Arabian	15 (19,0%)
African/afroamerican	5 (6,3%)
**Treatment with ESAs**, ***n*** **(%)**	32 (40.5%)
**Total FCM dose (mg)**	
Median (P25–P75)	500 (280–500)
**FCM dose (mg/kg)**	
Median (P25–P75)	15.4 (12.7–18.5)
**Total number of doses**	131
**Number of doses per patient**, ***n*** **(%)**	50 (63%) 13 (16%)
1 dose	
2 doses	16 (20%)
>2 doses	50 (63%)
**Duration of infusion, min**	
Median (P25–P75)	60 (45–60)
Media (SD)	62,1 (23,76)
**Diagnoses, N**	
Glomerulopathies	28
CAKUT	14
Hereditary cystic KD (ciliopathy)	11
Metabolic nephropathy	10
Tubulopathy	6
KD due to vascular causes	6
Thrombotic microangiopathy	4

CAKUT, congenital abnormalities of kidney and urinary tract; eGFR, estimated glomerular filtration rate; ESAs, erythropoiesis–stimulating agents; FCM, ferric carboxymaltose; min, minutes; P25–P75, interquartile range (25th percentile to 75th percentile); IV, intravenous; KD, kidney disease; n, number of patients.

**Table 2:** Patients CKD stage^*^.

**Glomerular filtration rate category (mL/min/1.73 m** ^**2**^ **)**		**Number of patients**
**Description**	**Range**	n = 76^**^
G1	> 90	28
G2	60–89	22
G3a	45–59	8
G3b	30–44	5
G4	15–29	10
G5	<15	3
PD	–	6

PD, peritoneal dialysis.

* According to the chronic kidney failure classification of the KDIGO 2012 guidelines (3).

** eGFR data were obtained from 76/79 patients.

All patients exhibited good tolerability to FCM infusions, and no serious hypersensitivity reactions or anaphylactic reactions were observed. Only one adverse event was identified in a 16-year-old patient, who experienced extravasation at the end of the infusion. At the time it was detected, it was treated with Burow's solution, local cold, and limb elevation. The patient developed painless discoloration in the affected area of the anterior side of the forearm, with subsequent partial improvement.

Pre- and post-infusion Hb data were obtained in 58/79 (73.4%) patients. A total of 70.7% (41/58) of patients had anemia at the initiation of treatment according to the normal Hb range for their age. The rest of the patients had adequate Hb levels but low serum iron deposits with TS ≤ 20% and/or ferritin ≤ 100 ng/mL. Positive response to FCM was observed in most patients (Table 3). Further, ferritin and TS normalization was observed after FCM infusion in 35/39 and 21/31 patients for whom data were available.

**Table 3:** Pre– and post–infusion changes in ferric carboxymaltose (FCM).

**Laboratory parameter**	**Baseline**	**30 days (±15)**	Overall change^*^
***Hemoglobin, g/dL***			
*n*	58	52	1.4 (0.9–1.9)
Median (P25–P75)	10.4 (9.1–11.7)	11.8 (10.5–13.2)	
without ESAs			
*n*	31	28	
Median (P25–P75)	11.4 (9.8–12.2)	12.6 (11.4–13.3)	1.3 (0.7–1.7)
with ESAs			
*n*	27	24	
Median (P25–P75)	10.4 (8.8–10.9)	11.4 (10.2–12.3)	1.6 (1.1–2.8)
***Ferritin (ng/mL)***			
*n*	57	43	
Median (P25–P75)	32.0 (16–68)	285 (169.5–409.5)	224 (136–378.5)
***TS (%)***			
*n*	53	40	
Median (P25–P75)	11 (7–15)	31 (20.8–38.5)	18 (9.3–27.8)
***Fe (μg/dL)***			
*n*	55	44	
Median (P25–P75)	28 (21–40)	66 (50.8–101.8)	37 (17.5–71)

Fe, serum iron; n, number of patients; P25–P75, interquartile range (25th percentile to 75th percentile).

* For each patient, changes in pre–infusion (baseline) and post–infusion laboratory results were based on the highest value of the specific laboratory result if there were multiple values for the same period.

Reference range: Hb (anemia if ≤ 11.0 g/dL in children aged 0.5–5 years, ≤ 11.5 g/dL in children aged 5–12 years, ≤ 12 g/dL in children aged 12–15 years, and ≤ 12 g/dL or ≤ 13 g/dL in adolescent girls or boys over 15 years old, respectively; ferritin (25–400 ng/mL); TS (20–55%); Fe (50–150 μg/dL).

Table 3 lists Hb variations for all patients who had an increase in Hb or maintained a stable value (89.7%), and the stratified data are described according to whether they were receiving ESAs or not.

Only in 6 (10.3%) patients, FCM administration was not associated with anemia improvement during the study period. Two of them, experienced significant intercurrent infections at that time (1 mumps and 1 urologic sepsis, respectively) that required hospitalization and possibly explained lack of response. Apparently, no specific cause apart from the underlying disease or treatment, was observed in additional four patients who did not present Hb increase, although in all them ferritin and/or TS increased after FCM.

The median baseline phosphate concentration was 4.9 mg/dL (4.3–5.3). Post-infusion phosphate values were obtained for 43 patients (79.3%), with a median decrease of 0.5 mg/dL (0.1–1.1) observed in 22 (51.2%) patients. No patients developed low blood phosphate levels according to the normal age-adjusted values.

Table 3 summarizes laboratory tests variation at baseline and after treatment.

## Discussion

Most of published retrospective observational studies on the use of FCM in children include patients with gastrointestinal conditions, followed by metrorrhagia-related anemia (2, 5, 6, 9, 13–17). Our study is the first to describe the effectiveness and safety of FCM treatment in a series of 79 pediatric patients with kidney disease non-dependent on hemodialysis.

Lass et al. (9) analyzed a population of 72 pediatric patients with iron-deficiency anemia who received FCM, observing mild hives and/or edema in three patients. Another series described by Powers et al. (13) with 72 patients who had inflammatory bowel disease and anemia reported adverse effects in 7 (9.7%) patients who received FCM: 1 patient experienced dyspnea requiring respiratory support, corticosteroids, and diphenhydramine that resolved within 5 min, 1 patient experienced tingling at the infusion site, and 1 patient had extravasation of the medication. All other patients had mild hives or pruritus, which were treated with antihistamines and/or corticosteroids. Other authors have reported itching, hives on the trunk and legs, and low-grade fever in 2 (2%) of 101 patients after FCM was administered (3) and the appearance of mild rash in 2 (5.7%) of 35 patients (18).

Compared with other authors, our case series observed a very favorable safety and tolerability profile for the medication, with extravasation observed in only 1 patient at the end of FCM infusion.

However, the hematologic response showed a median increase in hemoglobin of 1.4 g/dL (0.9–1.9) in most cases (89.7%) after a median of 1 month, and an increase in ferritin and TS. We cannot exclude that a longer examination time of the study could have demonstrated a better response.

As observed in other studies, patients who received ESAs had lower baseline Hb values, and the observed Hb increase was larger than in those who did not receive ESAs (19). Patients with kidney disease and iron deficiency are known to require higher doses of ESAs (19), and the administration of IV iron increases sensitivity to ESAs, making it possible to lower ESA dosages (20, 21). In our study, we did not evaluate, the impact on the ESA dose prescribed due its retrospective design and the fact that the study aim was to evaluate tolerance and effectiveness of FCM on the short term.

Although oral iron is considered the treatment of choice in pediatric patients (except for specific cases such as hemodialysis) due to its non-invasiveness and lower cost, it is often poorly tolerated, is accompanied by absorption problems, and requires 2 to 3 weeks to raise hemoglobin concentrations and up to 6 months to achieve normal iron deposits (3). Nausea, abdominal pain, and constipation are AEs that affect adherence to oral iron therapy among the pediatric population. Because the FCM is a stable complex, this allows a maximum cumulative weekly dose of 1,000 mg of iron to be administered and fewer infusions are needed to administer the total iron dosage needed for each patient, compared with other formulations such as iron sucrose. In addition, the infusion time required ( ≤ 15 min) is lower than for iron sucrose (30 min to 2 h) (22), reducing day hospital occupancy levels.

Consequently, because the use of intravenous iron avoids intestinal absorption, gastrointestinal tolerance can be improved and the number of oral treatments these patients often receive can be decreased, making it an option with cost-saving potential for outpatients who only visit the hospital to receive intravenous iron (8). Greater work-life balance might be achieved for patients and their families by scheduling infusions at the same time as blood draws for lab work and hospital visits.

Good experience in terms of effectiveness and tolerability has made it possible to change the therapeutic approach taken with iron-deficient chronic patients eligible for hospital laboratory follow-up who require periodic intravenous treatment, an approach that has been well accepted by patients and their families.

The main limitation of this study was its observational and retrospective nature, making it impossible to obtain all laboratory data for the patients included.

In conclusion, these data provide evidence that FCM is a safe and effective therapeutic option in children based on the low incidence of adverse effects and the improvement of laboratory results.

## Data availability statement

The raw data supporting the conclusions of this article will be made available by the authors, without undue reservation.

## Ethics statement

The studies involving human participants were reviewed and approved by Clinical Research Ethics Committee of our hospital on December 20, 2019 under Protocol Code FCM-NEFRO-PED. Written informed consent from the participants' legal guardian/next of kin was not required to participate in this study in accordance with the National Legislation and the Institutional Requirements.

## Author contributions

PG-O organized the database and wrote the first draft of the manuscript. IJ-L contributed to design of the study and wrote sections of the manuscript. IJ-L, AP, ÁC, GA, and ML contributed to manuscript revision. All authors read and approved the submitted version.

## Funding

Funding was received from Vifor Pharma Spain for translation of the manuscript and support for an Open Access license. This did not influence the results obtained in this study because all data interpretation and manuscript reviews were carried out independently from the source of funding.

## Conflict of interest

The authors declare that the research was conducted in the absence of any commercial or financial relationships that could be construed as a potential conflict of interest.

## Publisher's note

All claims expressed in this article are solely those of the authors and do not necessarily represent those of their affiliated organizations, or those of the publisher, the editors and the reviewers. Any product that may be evaluated in this article, or claim that may be made by its manufacturer, is not guaranteed or endorsed by the publisher.
